# A Rare Finding in Pregnancy

**DOI:** 10.1016/j.acepjo.2026.100470

**Published:** 2026-07-17

**Authors:** Nicholas Pigg, Gili Edry, Daniel Ridelman

**Affiliations:** 1 Department of Emergency Medicine, Wayne State University School of Medicine, Detroit, Michigan, USA; 2 Department of Radiology, Wayne State University School of Medicine, Detroit, Michigan, USA

## 1 Patient Presentation

A 31-year-old primigravid women with past medical history of multiple sclerosis and uterine fibroids was referred to the emergency department by her obstetrician for evaluation of a possible twin ectopic pregnancy found on a routine outpatient ultrasound. She was asymptomatic, denying any pelvic pain or vaginal bleeding. By the last menstrual period, she was 6 weeks pregnant. She was not on fertility treatment. Her physical examination was unremarkable. Serum human chorionic gonadotropin was 22,606 mIU/mL. Transabdominal and transvaginal obstetric ultrasound visualized no intrauterine pregnancy.

## 2 Diagnosis: Triplet Ectopic Pregnancy

**Figure 1:**
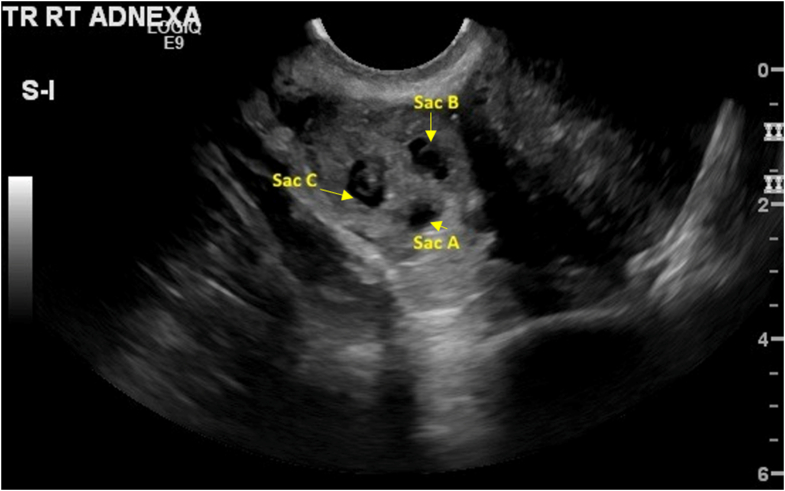
Triplet gestational sacs in the right adnexa. Sac A measured 0.64 cm, corresponding to a gestational age of 5 weeks and 2 days, and had no discernable yolk sac or heart rate.

**Figure 2:**
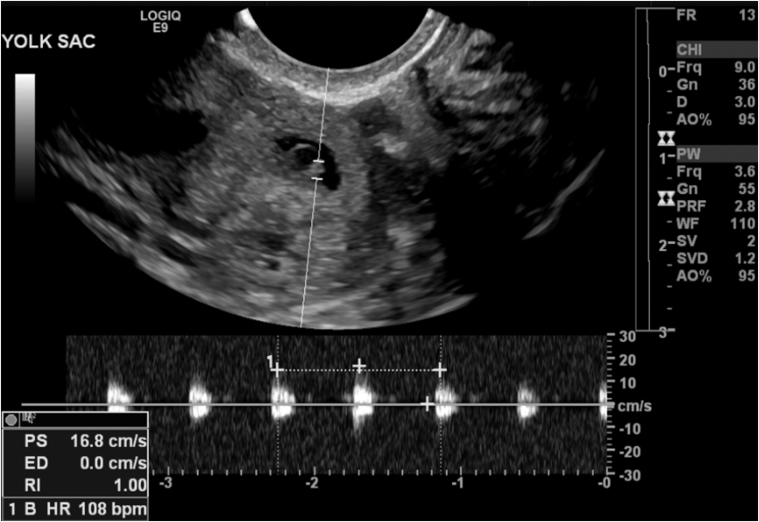
Sac B measured 0.69 cm, corresponding to a gestational age of 5 weeks and 3 days, and had a heart rate of 108 bpm.

**Figure 3:**
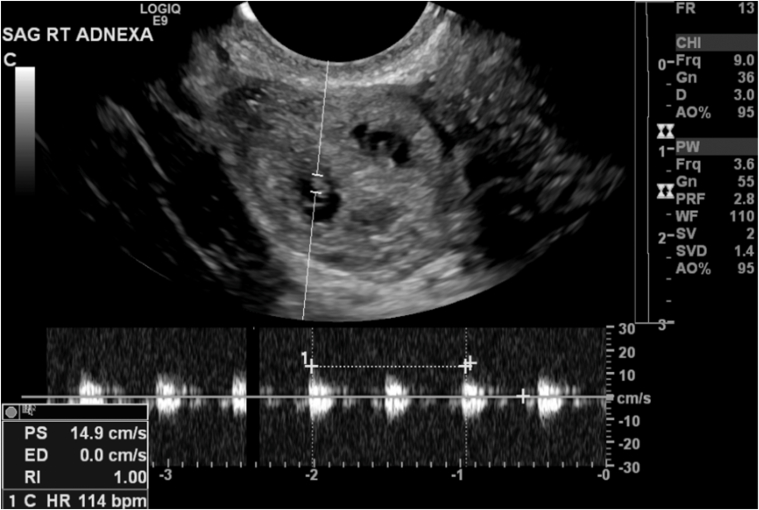
Sac C measured 0.77 cm, corresponding to a gestational age of 5 weeks 6 days, and had a heart rate of 114 bpm.

Ultrasound imaging revealed 3 unruptured gestational sacs in the right adnexa, 2 of which had yolk sacs, fetal poles, and discernible heart rates (Figure 1, Figure 2, Figure 3). Unfortunately, the patient left prior to the interpretation of her ultrasound. She returned 2 days later and underwent exploratory laparoscopy. She was found to have extensive and complex intraabdominal adhesions and “violin string” hepatic adhesions, consistent with Fitz-Hugh-Curtis syndrome. After extensive lysis of adhesions, the patient underwent right salpingo-oophorectomy and successful removal of the triplet ectopic pregnancies. She was discharged on the same day.

Pulsed wave and doppler imaging ultrasound were used to increase the diagnostic yield in this case because the pregnancies were determined to be nonviable.

To the authors’ knowledge, this would be the 8th reported case of spontaneous triplet ectopic pregnancy described in the literature. Although extremely rare, this is an important ultrasound finding that should not be confused with a normal ovary.

## Conflict of Interest

All authors have affirmed they have no conflicts of interest to declare.

